# Hip geometric parameters are associated with radiographic and clinical hip osteoarthritis: findings from a cross-sectional study in UK Biobank

**DOI:** 10.1016/j.joca.2023.09.001

**Published:** 2023-09-11

**Authors:** SV Heppenstall, R Ebsim, FR Saunders, C Lindner, JS Gregory, RM Aspden, NC Harvey, T Cootes, JH Tobias, M Frysz, BG Faber

**Affiliations:** 1 Musculoskeletal Research Unit, University of Bristol, UK; 2 Division of Informatics, Imaging and Data Sciences, The University of Manchester, UK; 3 Centre for Arthritis and Musculoskeletal Health, University of Aberdeen, UK; 4 Medical Research Council Lifecourse Epidemiology Centre, University of Southampton, UK; 5 NIHR Southampton Biomedical Research Centre, University of Southampton and University Hospital Southampton NHS Foundation Trust, UK; 6 Medical Research Council Integrative Epidemiology Unit, University of Bristol, UK

**Keywords:** DXA, epidemiology, hip osteoarthritis, hip geometry, hip shape

## Abstract

**Objectives:**

To examine the extent to which geometric parameters derived from dual-energy x-ray absorptiometry (DXA) scans in the UK Biobank study are related to hip osteoarthritis (HOA) independently of sex, age and body size.

**Design:**

Femoral neck width (FNW), diameter of the femoral head (DFH) and hip axis length (HAL) were derived automatically from left hip DXA scans in UK Biobank using outline points placed around the hip by a machine-learning program. Correlations were calculated between geometric parameters, age, height, and weight. Logistic regression was used to examine the relationship of geometric parameters with radiographic hip osteoarthritis (radiographic HOA), and hospital diagnosed HOA (HESOA), and Cox proportional hazards model to evaluate the relationship with total hip replacement (THR). Analyses were adjusted for sex, age, height, weight, and geometric parameters.

**Results:**

The study consisted of 40,312 participants. In age and sex-adjusted analyses, FNW, HAL and DFH were related to increased risk of radiographic HOA. In a model adjusted for age, sex, height, weight and other geometric parameters, both FNW and HAL retained independent relationships with radiographic HOA [FNW: OR 2.38 (2.18-2.59), HAL: 1.25 (1.15-1.36)], while DFH was now protective [0.55 (0.50-0.61)]. Only FNW was independently related to HESOA [2.20 (1.80-2.68)] and THR [HR 2.51 (1.89-3.32)].

**Conclusion:**

Greater FNW and HAL were independently related to an increased risk of radiographic HOA, whereas greater DFH appeared to be protective. Greater FNW was independently associated with HESOA and THR. These results suggest DXA-derived geometric parameters, particularly FNW, could help determine HOA and THR risk.

## Introduction

Osteoarthritis (OA) is a major cause of pain and disability globally, with the hip being the third most commonly affected joint (1). Morphological variation in hip shape has long been postulated as a risk factor for the development of hip OA (HOA) (2–5). To explore these associations, geometric parameters of hip shape have been measured on 2-dimensional imaging. Thus, femoral neck width (FNW), hip-axis length (HAL) and diameter of the femoral head (DFH) have been shown to associate with HOA when examined individually in small studies, but the inter-relatedness of these measures have not been explored previously (6, 7).

Hip shape is known to vary greatly between the sexes with females having a larger neck shaft angle and smaller femoral head and neck (8, 9). However, geometric parameters measuring femur size are intrinsically related to body size and each other (6). This has made understanding independent associations between geometric parameters and HOA difficult especially given previous studies have tended to examine aspects of shape in isolation without sex stratification (6, 7). Statistical Shape Modelling (SSM) has evolved as an alternative approach to quantifying hip morphology and captures the whole of the hip joint. This method does overcome the issue of geometric parameters being correlated with size and each other, however the main limitation of SSM is that it is challenging to determine which specific aspects of hip shape are related to the outcome of interest. Therefore, geometric parameters can provide complementary information, hence why we have decided to look at them separately.

The availability of large cohorts with hip dual-energy X-ray absorptiometry (DXA) scans linked to HOA outcomes, such as in the UK Biobank study, provides an excellent opportunity to examine relationships between geometric parameters and HOA in more detail. Improvements in scan resolution have shown images acquired with newer DXA scanners to be suitable for ascertaining both hip shape and radiographic HOA measures (10, 11). In addition, DXA scans involve lower doses of radiation than traditional radiographs, offering the potential for use in screening. Existing methods such as hip structural analysis (HSA) are already available for deriving geometric parameters such as FNW and HAL for hip DXA scans (12), however this does not generate other parameters potentially related to HOA such as DFH.

The UK Biobank study has undertaken ~40,000 high resolution hip DXA scans. This large sample now offers opportunities to explore relationships between geometric parameters and HOA as defined both clinically and radiographically. To understand the relationship between geometric parameters and HOA, in the present study, we aimed to: (i) determine the correlation between geometric parameters and measures of body size, (ii) describe the cross-sectional relationships between geometric parameters with radiographic HOA and hospital diagnosed hip OA (HESOA), (iii) describe the longitudinal associations between geometric parameters and total hip replacement (THR) and (iv) establish which of these are independent as assessed in mutually adjusted models.

## Materials and Methods

### Population

UK Biobank is a large prospective study, which at baseline (2006-2010), recruited over 500,000 men and women aged 40-69 years (13). Participants have undergone extensive phenotypic assessments through questionnaires, imaging, physical measures, and electronic healthcare record linkage (14). In 2014 UK Biobank commenced the extended imaging study with the aim of conducting DXA scans on 100,000 participants but as of the start of this study (November 2022) ~40,000 were available. DXA scans were obtained from both hips (iDXA GE-Lunar, Madison, WI), with participant’s limbs being positioned with 15-25° internal rotation using a standardised protocol. UK Biobank has ethical approval from the National Information Governance Board for Health and Social Care and North-West Multi-centre Research Ethics Committee (11/NW/0382) which covers this study (application number 17295). All participants gave informed written consent.

### Dual-energy X-ray absorptiometry variables

#### Outline points and radiographic osteoarthritis annotation

**Figure 1:**
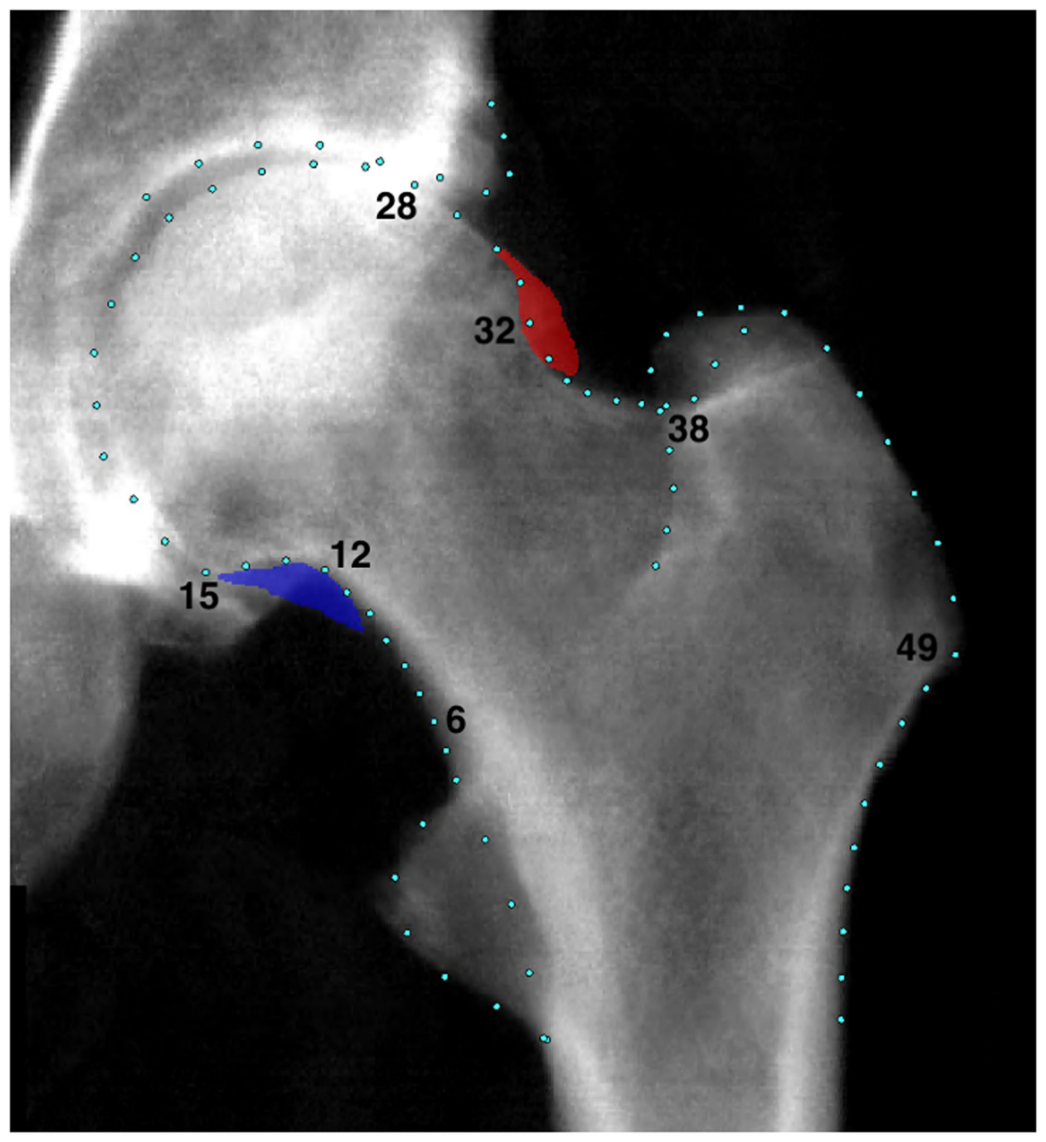
An example of a grade 4 rHOA hip from UK Biobank with superolateral (red) and inferomedial (blue) osteophytes marked.

A machine-learning algorithm placed 85 points to outline the proximal femur and acetabulum in all available left hip DXA scans as of April 2021 (11). Each image was checked, and points were corrected if necessary (~90% of images required no correction) and at the same time osteophytes were manually annotated using a custom tool (The University of Manchester) (Figure 1). The outline points did not encompass any annotated osteophytes. Reproducibility of the point placement is high (kappa 0.93) as previously reported (10). Radiographic HOA grades 0-4 that have been previously validated against clinical outcomes were assigned semi-automatically to each hip DXA image combining osteophyte and joint space width data which have a good reproducibility (intra-rater kappa’s between 0.80-0.93) (10).

#### Geometric parameters

**Figure 2:**
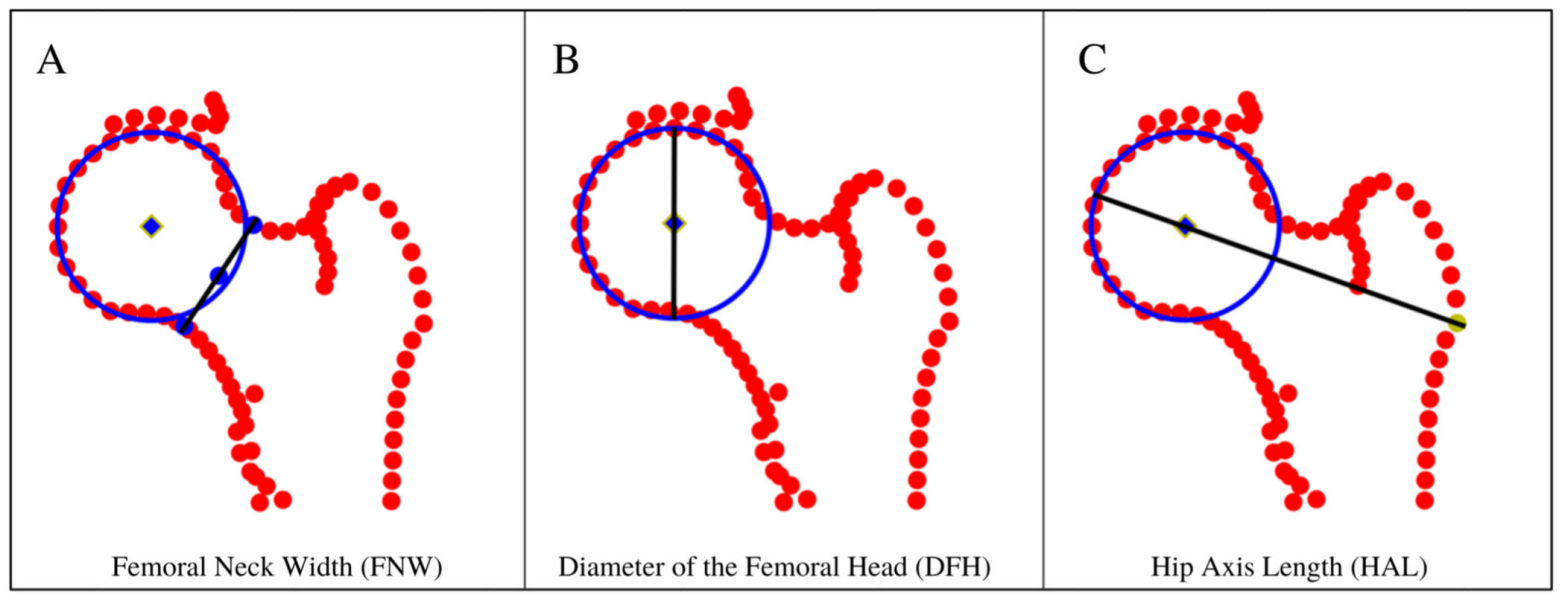
Geometric parameter measurements: A - Femoral Neck Width (FNW) derived using the line-segment method using points 6-12 on the medial side and points 32-38 on the lateral side, B - Diameter of the femoral head (DFH) derived by fitting a circle to points 15-28, C - Hip Axis Length (HAL) derived by finding the distance between point 49 and where the line intersects the circumference of the circle having passed through the centre of the femoral head.

Custom Python 3.0 scripts were developed and used to automatically derive FNW, DFH and HAL. These scripts are openly available (15). The DXA DICOM images store pixel dimension data facilitating the calculation of geometric parameters in millimetres (mm). FNW was defined as the shortest distance measured between the superior and inferior side of the femoral neck (16). To measure this, points 6-12 defined the inferior side and points 32-38 defined the superior side of the femoral neck (Figure 1). A line-segment approach was used to automatically calculate the narrowest distance between these points (Figure 2A), a description of this approach has been published previously (17). DFH was defined as the distance across the spherical aspect of the femoral head. To estimate this, a circle of best fit was placed around the femoral head using a least-squares package in Python that was applied to points 15-28 (18). The diameter of the circle was taken to represent the DFH in millimeters (Figure 2B). HAL was defined as the distance from the base of the greater trochanter to the medial aspect of the femoral head in millimeters. In previous studies, HAL measured using HSA software included medial joint space width as it is measured to the inner pelvic rim (19). In this study however, outline points were not reliably available for the medial acetabulum hence the measure only encompassed the femur. To measure this, a straight line was drawn from point 49 through the centre of the circle of best fit (used to calculated DFH). HAL was calculated from point 49 to where the line intersected the circumference of the circle after it has passed through the centre point of the circle (Figure 2C). The Python scripts were rigorously tested against manual measures in their creation and once applied to the dataset all images with values +/- 3 standard deviations (SDs) from the mean were reviewed manually confirming the appropriate measurements had been made. In addition, FNW and HAL measures derived automatically were compared with values derived from HSA software (iDXA GE-Lunar, Madison, WI) in a subset of participants to check comparability.

#### Clinical outcomes

HESOA and THR data were obtained from hospital episode statistics (HES) collected until 31^st^ December 2020. HESOA describes prevalent HOA and includes individuals with a hospital diagnosis of HOA before or after their DXA scan. Whereas THR describes incident procedures that happened only after their DXA. Further information on how these data were derived is available in Supplementary Methods and has been described previously (10). The clinical outcomes used in this study were not side-specific.

#### Statistical analysis

Baseline characteristics are shown as means, SDs and ranges for continuous variables and as frequencies for categorical variables. Correlations between geometric parameters, height, weight, and age were derived using Pearson’s correlation test statistic (r^2^). Distributions of continuous variables were checked visually for normality (Supplementary Figure 1). Frequencies were used for categorical variables. Correlation values ranging between r^2^ ≥ 0.7-1.0, r^2^ ≥ 0.5-0.7, and r^2^ < 0.5 were deemed as strong, moderate and weak correlations respectively. Logistic regression was used to examine associations between each standardised exposure (FNW, DFH and HAL) and each HOA outcome (grade ≥2 radiographic HOA and HESOA). Assumptions of linearity were checked using Box-Tidwell tests. Sensitivity analyses were carried out using grade ≥3 radiographic HOA and grade 4 radiographic HOA only as outcomes. Results are presented as odds ratios (OR) with 95% confidence intervals (CIs) and p values. Cox proportional hazard modelling was used to investigate the associations between standardised geometric parameters and THR and results are presented as hazard ratios (HR) with 95% CIs and p values. The Cox proportional hazard and linearity assumptions were tested using log-log plots and Martingale residuals respectively. We censored for deaths that occurred during the study period [n=368 (0.9%)]. Due to the availability of hospital episode statistics, the study end date was 31^st^ December 2020. We present unadjusted (model 1), and confounder adjusted analyses (partially adjusted = models 2, 3 and fully adjusted = model 4). Potential confounders were defined *a priori*, and these were included in partially adjusted models as covariates (model 2: adjusted for age and sex; model 3: model 2 plus height and weight to account for effects of body size with which geometric parameters are known to be strongly related) (6, 20–22). A final model also included mutual adjustment for other geometric parameters (fully adjusted/model 4). Sex- stratified analyses were conducted due to known differences in hip shape. All statistical analyses were performed using STATA version 17 (Stata Corp, College Station, TX, USA). Furthermore, a glossary of terms is provided for the reader in Supplementary Table 1.

## Results

### Baseline characteristics

A total of 40,312 individuals (mean age 63.7, SD 7.6 years, range 44-82) had left hip DXAs available for analysis (Table 1). 21,021 (52.1%) participants were women and 19,291 (47.9%) were men. radiographic HOA grade ≥2 was present in 3,014 (7.5%) individuals, radiographic HOA grade ≥3 in 700 (1.7%) and radiographic HOA grade 4 in 157 (0.4%). Males had a higher mean weight (male 83.2kg versus female 68.2kg) and prevalence of radiographic HOA across all three grades compared with females. 527 (1.3%) individuals had HESOA and 259 (0.6%) underwent THR after their hip DXA. In contrast with radiographic HOA, the prevalence of clinical outcomes was higher in females compared with males (Table 1).

### Geometric parameters and their inter-relationships

The mean FNW was 31.6 mm (SD 3.5, range 21.4- 45.8), DFH 45.9 mm (3.8, 33.4-64.4) and HAL 96.7 mm (8.0, 68.1-127.1). Males had a greater FNW, DFH and HAL compared with females [FNW: male mean 34.5 mm (SD 2.4, range 22.9-45.8) / female mean 29.0 mm (SD 2.0, range 21.4-37.8), DFH: 49.0 mm (2.6, 34.7-64.4)/ 43.0 (2.3, 33.4-53.7) & HAL: 103.1 mm (5.5 76.9-127.1)/ 90.8 mm (4.8, 68.1-115.5)] (Table 1). Comparison with HSA derived FNW and HAL showed strong correlations between these measures (r^2^ 0.97 & 0.93 respectively), however the mean values derived with HSA were larger (Supplementary Table 2). FNW, DFH, HAL and height were all strongly correlated (r^2^ 0.75-0.89) (Table 2). Weight also showed moderate correlations with FNW, DFH, HAL and height (r^2^ 0.52-0.57).

### Femoral neck width versus HOA

**Figure 3:**
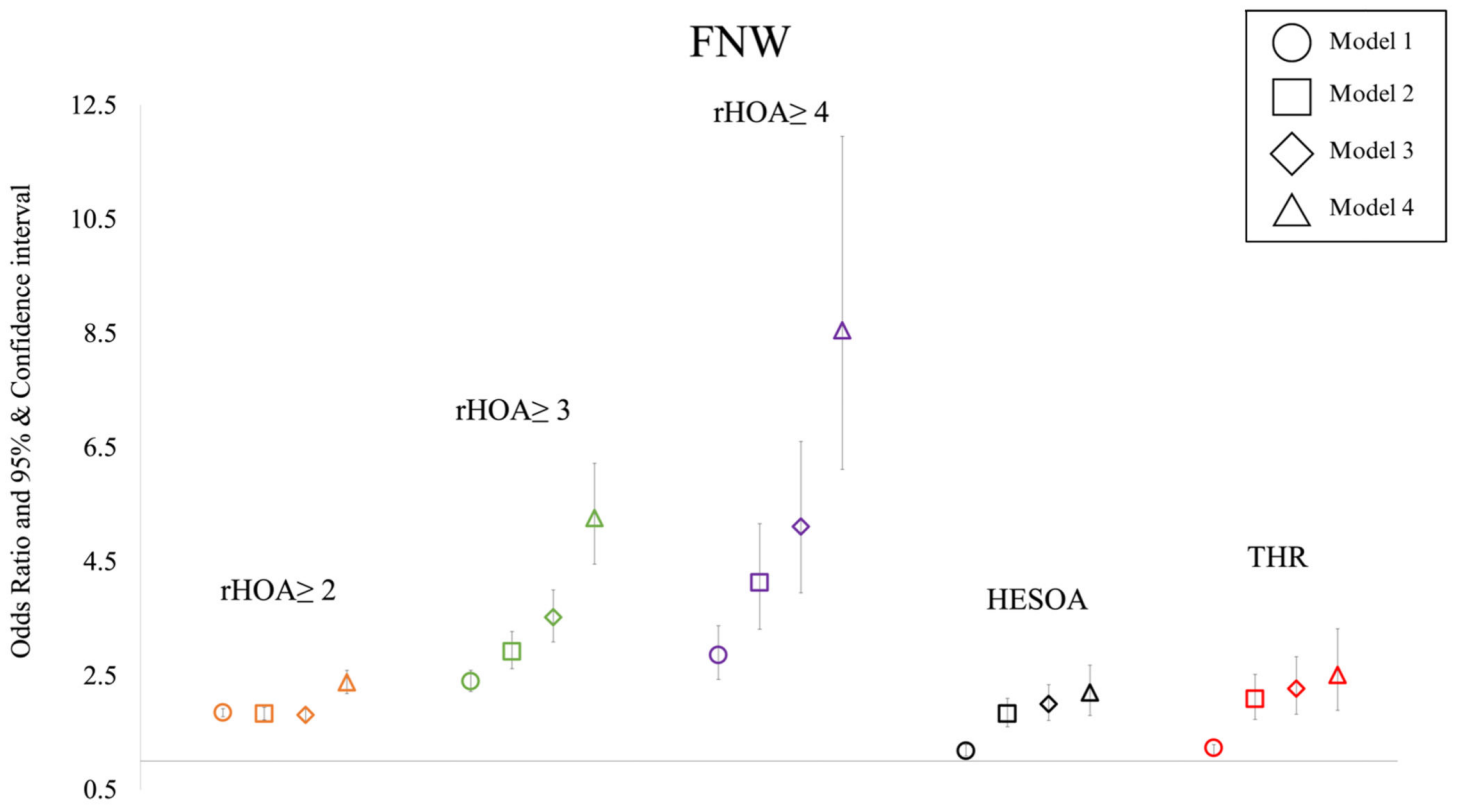
Logistic regression results for the associations between Femoral neck width (FNW) and radiographic hip osteoarthritis ≥ grade 2, grade ≥ 3, grade 4, hospital diagnosed OA (HESOA) and total hip replacement (THR) in sex-combined analyses. Each symbol represents odds ratios with 95% CIs. Square symbol indicates adjustment for age and sex, diamond for age, sex, height and weight and triangle for age, sex, height, weight, and remaining GPs.

In analyses adjusted for age, sex, height and weight (model 3) progressive associations between a wider FNW and higher radiographic HOA grades were seen [radiographic HOA grades ≥2: OR 1.81 (95% CI 1.70-1.94), grades ≥3: 3.52 (3.09-4.00) and grade 4: 5.11 (3.95-6.60)]. Similar results were also seen in unadjusted (model 1) and age and sex adjusted (model 2) analyses (Supplementary Table 3). If anything, associations with radiographic HOA were strengthened when adjusting for sex, height and weight as well as geometric parameters (model 4) [grade ≥2 radiographic HOA: OR 2.38 (95% CI 2.18-2.59), grade ≥3 radiographic HOA: 5.26 (4.45-6.22) and grade 4 radiographic HOA: 8.55 (6.11-11.95)] (Figure 3, Table 3). Further sex stratified analyses showed similar associations in females and males albeit female effect estimates tended to have wider confidence intervals (Supplementary Figure 2, Supplementary Tables 4 and 5). Partially adjusted sex combined analyses (model 3) showed an association between FNW and HESOA [OR 2.00 (95% CI 1.71-2.34)] and THR [HR 2.27 (95% CI 1.82-2.83)]. These associations were strengthened after adjusting for other geometric parameters [model 4: HESOA: OR 2.20 (95% CI 1.80-2.68), THR: HR 2.51 (95% CI 1.89-3.32)]. (Figure 3, Table 3).

### Hip axis length versus HOA

**Figure 4:**
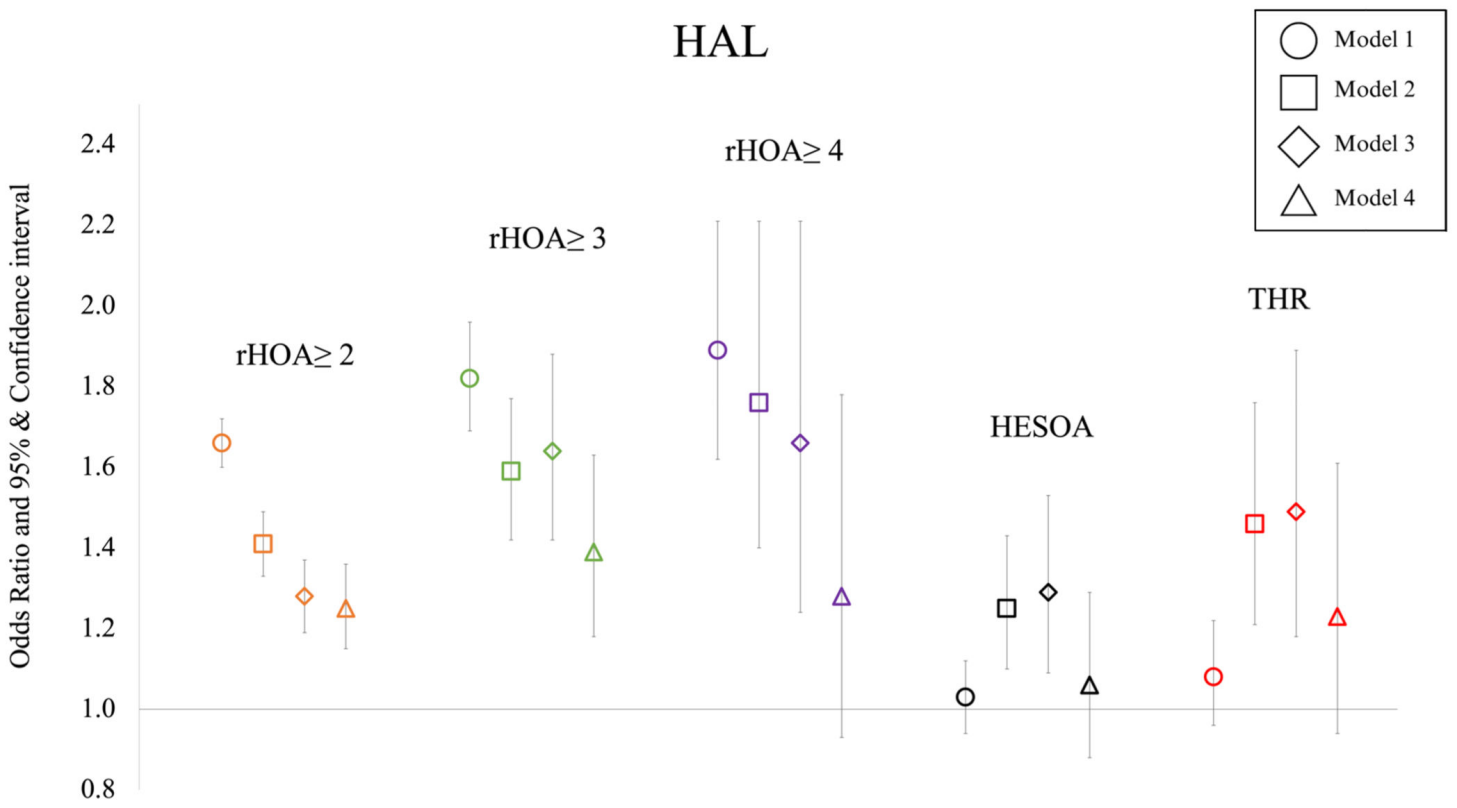
Logistic regression results for the associations between hip axis length (HAL) and radiographic hip osteoarthritis ≥ grade 2, grade ≥ 3, grade 4, hospital diagnosed OA (HESOA) and total hip replacement (THR) in sex-combined analyses. Each symbol represents odds ratios with 95% CIs. Square symbol indicates adjustment for age and sex, diamond for age, sex, height and weight and triangle for age, sex, height, weight, and remaining GPs.

In partially adjusted (model 3) sex combined analyses, there were associations between a longer HAL and higher radiographic HOA grades [radiographic HOA grades ≥2: OR 1.28 (95% CI 1.19-1.37), grades ≥3: 1.64 (1.42-1.88) and grade 4: 1.66 (1.24-2.21)]. These findings were consistent with unadjusted (model 1) and age and sex adjusted (model 2) analyses (Supplementary Table 3). On complete adjustment (model 4) there was some attenuation of effect between HAL and each radiographic HOA grade [radiographic HOA grades ≥2: OR 1.25 (95% CI 1.15-1.36), grades ≥3: 1.39 (1.18-1.63) and grade 4: 1.28 (0.93-1.78)] (Figure 4, Table 3). Sex stratified analyses showed stronger associations in females compared with males especially for the higher radiographic HOA grades [model 4: female/male radiographic HOA grade 4 OR 2.05 (95% CI 1.08-3.89) / 1.08 (0.74-1.57)] (Supplementary Figure 2, Supplementary Tables 4 and 5).

In partially adjusted (model 3) sex combined analyses there was some evidence of an association between HAL and HESOA and THR [HESOA: OR 1.29 (95% CI 1.09-1.53), THR: HR 1.49 (95% CI 1.18-1.89)]. After adjusting for other geometric parameters (model 4) these effect sizes were fully attenuated [HESOA: OR 1.06 (95% CI 0.88-1.29), THR: HR 1.23 (95% CI 0.94-1.61)]. (Figure 4, Table 3). Associations between HAL and HESOA and THR were broadly similar in the sex-stratified analyses (Supplementary Figures 3 and 4, Supplementary Tables 4 and 5).

### Diameter of the femoral head versus HOA

**Figure 5:**
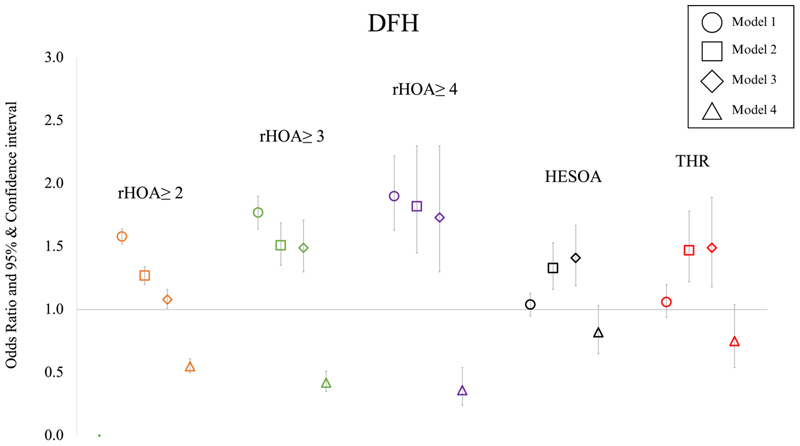
Logistic regression results for the associations between diameter of the femoral head (DFH) and radiographic hip osteoarthritis ≥ grade 2, ≥ grade 3, grade 4, hospital diagnosed OA (HESOA) and total hip replacement (THR) in sex-combined analyses. Each symbol represents odds ratios with 95% CIs. Square symbol indicates adjustment for age and sex, diamond for age, sex, height and weight and triangle for age, sex, height, weight, and remaining GPs

An association between a greater DFH and higher radiographic HOA grades was present in partially adjusted (model 3) sex combined analyses [radiographic HOA grade ≥2: OR 1.08 (95% CI 1.01-1.16), grade ≥3: 1.49 (1.30-1.71) and grade 4 radiographic HOA: 1.73 (1.30-2.30)]. These effect sizes were reduced compared with the unadjusted (model 1) and age and sex adjusted (model 2) analyses (Supplementary Table 3). After adjusting for other geometric parameters, the direction of effect was reversed with increasing DFH displaying a protective effect with radiographic HOA [radiographic HOA grades ≥2: OR 0.55 (95% CI 0.50-0.61), grades ≥3: 0.42 (0.35-0.51) and grade 4: 0.36 (0.24-0.54)] (Figure 5, Table 3). Sex stratified analyses revealed similar results in females and males (Supplementary Figure 2, Supplementary Tables 4 and 5).

On partial adjustment (model 3) there was some evidence of an association of DFH with HESOA [OR 1.41 (95% CI 1.19-1.67)] and THR [OR 1.50 (95% CI 1.19-1.90)]. After adjusting for other geometric parameters (model 4) these associations attenuated [HESOA OR 0.82 (0.65-1.03)] and THR [HR 0.75 (95% CI 0.54-1.04)] (Figure 5, Table 3). Sex stratified results were similar to combined results in terms of magnitude and direction of effect (Supplementary Figures 3 and 4, Supplementary Tables 4 and 5).

### Discussion

This is the largest observational study to date (N=40,312) exploring the associations between hip geometric parameters (FNW, DFH and HAL) and HOA. As expected, the geometric parameters were highly correlated with height and each other, as well as differing between the sexes. FNW, HAL and DFH were all related to increased risk of radiographic HOA in age and sex adjusted analyses (model 2). Despite strong relationships between geometric parameters and height and weight, relationships between geometric parameters and HOA showed little attenuation once adjusted for height and weight. Following adjustment for geometric parameters (model 4) both HAL and FNW retained independent relationships with radiographic HOA, whereas DFH was now protective. When considering the clinical outcomes, only FNW was independently related to HESOA and THR. These analyses suggest that a wider FNW, longer HAL and smaller DFH were associated with higher risk of radiographic HOA. In contrast, only a wider FNW appeared to be associated with increased risk of HESOA and THR.

FNW showed the strongest associations with all HOA outcomes, consistent with findings from previous studies (6, 7, 23). In addition, these associations were strengthened when adjusted for body size (i.e., height and weight) and other geometric parameters which suggests that FNW is the leading geometric parameter associated with HOA, something which has not been shown before. Previous explanations for the association of FNW with HOA have focused on the mechanism of impingement of the widened femoral neck on the acetabulum akin to femoroacetabular impingement (23–25). An alternative explanation is that the femoral neck is widened as part of, or in parallel to the onset of HOA rather than the widened femoral neck causing HOA. Recent evidence from studies using SSM suggests a wider femoral neck is associated with more severe HOA and separately a genetic study suggested that a genetic predisposition to HOA led to cam-like changes at the femoral head (11, 26, 27). Both these studies suggest that hip shape is associated with but might not be a cause of HOA which has treatment implications.

DFH showed positive associations with HOA when considered in isolation, similar to a previous study (6), but when adjusted for the other geometric parameters the direction of effect reversed to show a relatively smaller femoral head was in fact a risk factor for radiographic HOA. One possible explanation is that a smaller femoral head results in a smaller contact area at the joint which increases the loading forces leading to biomechanical degeneration, however further research is needed to confirm the mechanism behind this (23).

That said, these results are in contrast to previous studies based on SSM which reported associations with HOA to have both a larger femoral head and neck (11, 28, 29). This might be explained by how DFH is defined; for example, with a spherical hip, the diameter across the femoral head is the same when measured on multiple axes. Whereas, on an aspherical (cam-type) femoral head the elliptical nature means that the transverse diameter is greater than the vertical. In this study, the circle of best fit was fitted to the medial aspect of the femoral head so as not to be distorted by cam-type femoral heads. This is not the case for the aforementioned SSM studies. The corollary of this might be that a smaller spherical femoral head is a risk factor for HOA alongside larger aspherical (cam-type) femoral heads.

Whilst there was evidence of an association between HAL and HOA outcomes, these associations were fully attenuated following adjustment. Previously, when assessed in isolation a study had shown associations with HOA similar to model 3 in this study i.e. without adjustment for other geometric parameters (6). These results highlight the importance of considering measures of hip morphology alongside each other when examining relationships with HOA, as well as body size. Height and weight are both known risk factors for HOA and were correlated with the geometric parameters featured in this study (30).

In this study we have used a new method based on SSM points to automatically derive geometric parameters from a large number of DXA scans. In contrast to previous HSA studies, we were able to use this method to derive DFH measurements. FNW, DFH and HAL were all larger in males in contrast to females which is consistent with previous studies showing body measures are on average greater in males than females (31, 32). The geometric parameters obtained in this study compared favourably to those obtained by HSA in terms of correlation, but this study showed smaller mean measures likely due to differences in calculating the geometric parameters (12). For example, this study calculates FNW as the shortest distance across the femoral neck whereas HSA derives the FNW from its area measure making direct comparison difficult. Also, the HAL definition in this study does not include the pelvis unlike HSA; a previous study found a mean HSA-derived HAL of 104.7 mm, slightly higher than the mean in this study (96.7 mm) (33). Comparing geometric parameters derived in this study with other studies provides further face validity for our methods; mean FNW in this study is similar to that reported in the female only Study of Osteoporotic Fractures (mean FNW 29.0 mm v 30.7 mm) (7). DXA-derived DFH was very similar to DFH measured on proximal femurs removed during hemiarthroplasty in a previous cross-sectional study (mean 45.9 mm vs 44.9 mm respectively) (34).

The strengths of this study include its large sample size obtained through novel automated measures which have facilitated the description of DXA-derived hip geometry in UK Biobank, including the derivation of DFH which is not provided by existing HSA software. A sample size of this magnitude paves the way for future use of genetic epidemiological methods, which can add further understanding of aetiology of hip shape and its role in the pathogenesis of HOA (26, 35). In addition, this study showed how hip geometric measures are longitudinally associated with THR justifying further work to understand whether these measures could be combined with measures of DXA-derived radiographic HOA to better understand THR risk. Furthermore, the use of DXA scans which require lower radiation exposure than X-rays makes them more desirable for screening (10, 36).

There are several limitations to this study. Firstly, this was an observational study, so we cannot infer causality. The UK Biobank database contains predominantly Caucasian participants, limiting generalisability of findings to other populations and warranting further research in more ethnically diverse settings. In addition, the clinical outcomes examined (HESOA and THR) were not side specific and this may have biased our effect sizes towards the null. It was not possible to calculate neck-shaft angle or femoral shaft width as the outline points did not extend distally below the lesser trochanter. That said, prior studies suggested that the three parameters which were included may have the greatest relevance for hip OA (6, 7, 23). Finally, although HSA is an established methodology for deriving hip geometry, we were keen to explore the role of DFH, which is not calculated by HSA. Therefore, we developed a bespoke platform for deriving FNW, HAL and DFH from 85 points outlining the proximal femur and acetabulum. Reassuringly, FNW and HAL generated by both methods correlated closely in a subset of participants.

In conclusion, three geometric parameters (FNW, DFH and HAL) were automatically derived from high resolution DXA scans in UK Biobank. Incorporating their inter-relatedness into regression models, strong independent associations were observed between FNW and HOAoutcomes (radiographic HOA, HESOA and THR). Weaker independent associations were seen between DFH and HAL, and radiographic HOA. This work paves the way for clinical translation as it suggests geometric parameters derived from DXA are associated with HOA, including the risk of future hip replacement, but further work is needed to understand how to combine measures of hip morphology to better understand HOA risk and progression.

## Supplementary Material

Supplementary Material

## Figures and Tables

**Table 1:** Population Characteristics

	Combined	Female	Male
	N=40,313	N=21,022	N=19,291
	Mean [SD, Range]	Mean [SD, Range]	Mean [SD, Range]
**Age at 2^nd^ follow up (years)**	63.7 [7.6, 44-82]	63.0 [7.4, 45-82]	64.3 [7.7, 44-81]
**Weight pre-DXA (kg)**	75.3 [15.1, 34-171]	68.2 [12.9, 34-169]	83.2 [13.4, 47-171]
**Height pre-DXA (cm)**	170.1 [9.4, 135-204]	163.6 [6.4, 135-196]	177.2 [6.6, 150-204]
**Narrowest femoral neck width (NNW) (mm)**	31.6 [3.5, 21.4-45.8]	29.0 [2.0, 21.4-37.8]	34.5 [2.4, 22.9-45.8]
**Diameter of the femoral head (DFH) (mm)**	45.9 [3.8, 33.4-64.4]	43.0 [2.3, 33.4-53.7]	49.0 [2.6, 34.7-64.4]
**Hip Axis Length (HAL) (mm)**	96.7 [8.02, 68.1-127.1]	90.8 [4.8, 68.1-115.5]	103.1 [103.1, 76.9-127.1]
	**N [%]**	**N [%]**	**N [%]**
**rHOA ≥ 2**	3,014 [7.5]	928 [4.4]	2,086 [10.8]
**rHOA ≥ 3**	700 [1.7]	191 [0.9]	509 [2.6]
**rHOA ≥ 4**	157 [0.4]	44 [0.2]	113 [0.6]
**Hip Pain > 3 months**	3,247 [8.0]	2,055 [9.8]	1,192 [6.2]
**Hospital diagnosed HOA (HESOA)**	527 [1.3]	307 [1.5]	220 [1.1]
**Total Hip Replacement (THR)**	259 [0.6]	153 [0.7]	106 [0.6]
**Duration from DXA to THR/ end of study (days)**	1173 [3 - 2437]	1171 [3-2436]	1175 [3-2437]

Abbreviations: FNW − Femoral Neck Width, DFH − Diameter of the Femoral Head, HAL − Hip Axis Length, rHOA − radiographic hip osteoarthritis, HESOA − Hospital Diagnosed Hip Osteoarthritis, THR − Total Hip Replacement

**Table 2:** Correlation (R^2^) matrix between geometric parameters, height, weight, and age.

GP	FNW	DFH	HAL	Height	Weight	Age
**FNW**	1					
**DFH**	0.89 (0.88-0.89)	1				
**HAL**	0.82 (0.81-0.82)	0.87 (0.87-0.87)	1			
**Height**	0.75 (0.75-0.76)	0.81 (0.80-0.81)	0.81 (0.80-0.81)	1		
**Weight**	0.55 (0.54-0.56)	0.52 (0.52-0.53)	0.53 (0.52-0.54)	0.57 (0.57-0.58)	1	
**Age**	0.13 (0.13-0.14)	0.11 (0.10-0.12)	0.13 (0.13-0.14)	-0.06 (-0.05--0.07)	-0.07 (-0.06--0.08)	1

Green indicates a strong correlation (r2 ≥ 0.7-1.0), yellow a moderate correlation (r2 ≥ 0.5- 0.7) and amber/red a weak correlation (r2 <0.5). Correlation coefficients are followed by 95% Confidence Intervals.

Abbreviations: GP − Geometric Parameter, FNW − Femoral Neck Width, DFH − Diameter of the Femoral Head, HAL − Hip Axis Length.

**Table 3:** Logistic regression/ Cox proportional hazard modelling results showing the association between geometric parameters and HOA outcomes.

		Grade ≥ 2 rHOA	Grade ≥ 3 rHOA	Grade ≥ 4 rHOA	HESOA	THR
		OR [95% CI]	OR [95% CI]	OR [95% CI]	OR [95% CI]	HR [95% CI]
Model 3	FNW	1.81 [1.70-1.94]	3.52 [3.09-4.00]	5.11 [3.95-6.60]	2.00 [1.71-2.34]	2.27 [1.82-2.83]
Model 4	FNW	2.38 [2.18-2.59]	5.26 [4.45-6.22]	8.55 [6.11-11.95]	2.20 [1.80-2.68]	2.51 [1.89-3.32]
Model 3	HAL	1.28 [1.19-1.37]	1.64 [1.42-1.88]	1.66 [1.24-2.21]	1.29 [1.09-1.53]	1.49 [1.18-1.89]
Model 4	HAL	1.25 [1.15-1.36]	1.39 [1.18-1.63]	1.28 [0.93-1.78]	1.06 [0.88-1.29]	1.23 [0.94-1.61]
Model 3	DFH	1.08 [1.01-1.16]	1.49 [1.30-1.71]	1.73 [1.30-2.30]	1.41 [1.19-1.67]	1.50 [1.19-1.90]
Model 4	DFH	0.55 [0.50-0.61]	0.42 [0.35-0.51]	0.36 [0.24-0.54]	0.82 [0.65-1.03]	0.75 [0.54-1.04]

Table shows logistic regression results for the associations between geometric parameters and grade ≥ 2,3 and 4 radiographic hip osteoarthritis (rHOA) and hospital diagnosed hip osteoarthritis (HESOA) and Cox proportional hazard modelling results between geometric parameters and total hip replacement (THR) in partially adjusted and fully adjusted combined sex analyses (model 3 included the covariates age, sex, height and weight whereas model 4 was adjusted for these as well as the remaining geometric parameters).
