# Supplementary material for: Hip geometric parameters are associated with radiographic and clinical hip osteoarthritis: findings from a cross-sectional study in UK Biobank
Source: Osteoarthritis Cartilage. Author manuscript; Available in PMC 2024 May 8. (PMC7615936; doi:10.1016/j.joca.2023.09.001)
Supplement: Supplementary Material [file EMS195520-supplement-Supplementary_Material_.docx]

**Supplementary Material**

**Supplementary Methods**

Hospital diagnosed osteoarthritis and total hip replacement data

Hospital diagnosed HOA (HESOA) was based on international classification of diseases (ICD) -9 &10 codes (a full list of codes included has previously been published (4)) released in hospital episode statistics (HES). HES data has been collected since 1981 in Scotland, 1997 in England and 1998 in Wales. This study examined data that was collected up until 31^st^ December 2020 which is the end point for our Cox proportional hazard models and linked to UKB in January 2021. Office of Population Censuses and Survey (OPCS) -3&4 codes are used to record operation procedures in HES, the codes included in this study were OPCS-3: 811, 810, OPCS-4: W371, W81, W391. These data were collected over the same period as described for HESOA and were linked to UKB in January 2021.

A total of 400/527 of the HESOA diagnoses took place after the DXA scan. Since 127 cases preceded the DXA scan we examined this variable cross-sectionally. In total, 259/260 THR happened after the DXA scan. The one participant with a THR predating the DXA scan had the procedure on the right side which was not imaged in this study as the left hip had a native hip imaged, this individual was excluded from the study. Hence THR was examined longitudinally using 259 cases.

**Supplementary Figures**


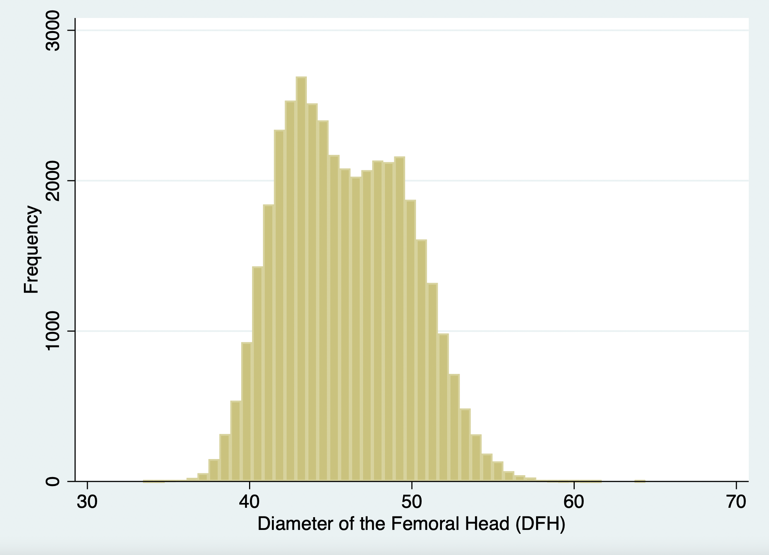

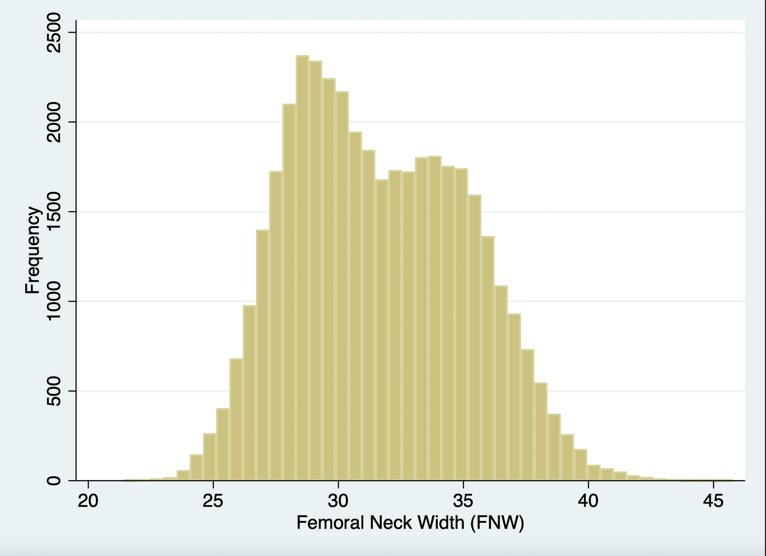

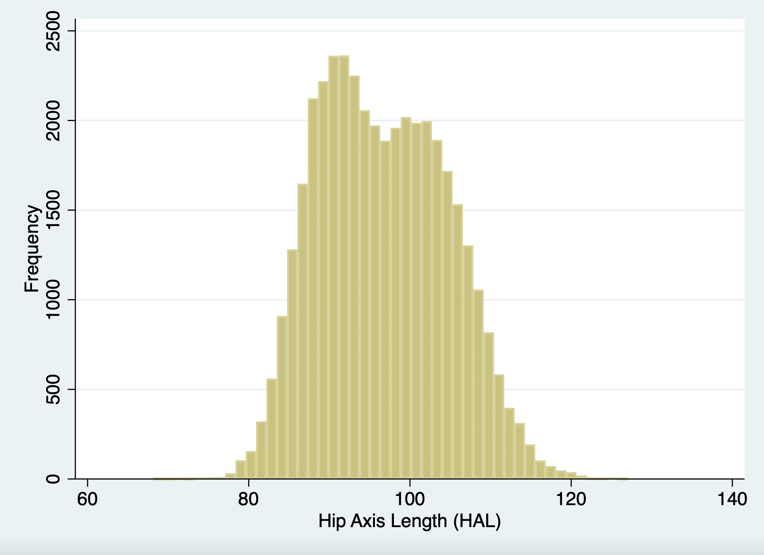


Supplementary Figure 1: Histograms demonstrating the distribution of data for Femoral Neck Width (FNW), Diameter of the Femoral Head (DFH) and Hip Axis Length (HAL).

**
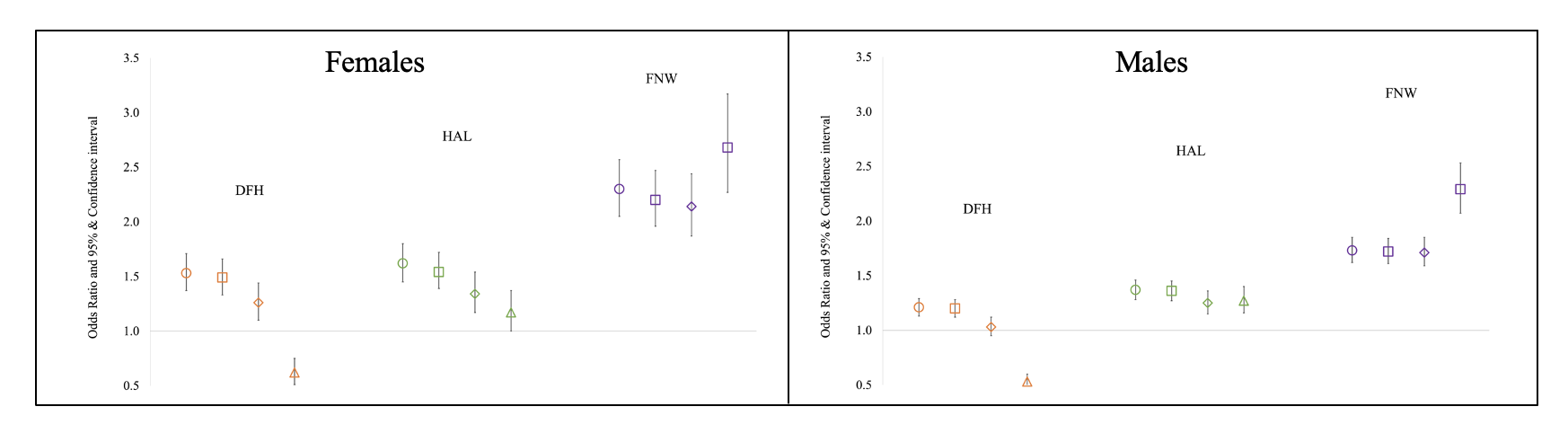
**

Supplementary Figure 2: Logistic regression results for the associations between geometric parameters – diameter of the femoral head (DFH), hip axis length (HAL) & femoral neck width (FNW) and radiographic hip osteoarthritis grade ≥ 2 in sex stratified analyses. Odds ratios with 95% CIs were plotted either side of the points. Circle symbol represents unadjusted analyses (model 1), square indicates adjustment for age and sex (model 2), diamond for age, sex, height and weight (model 3) and triangle for age, sex, height, weight, and remaining geometric parameters (model 4)*.*


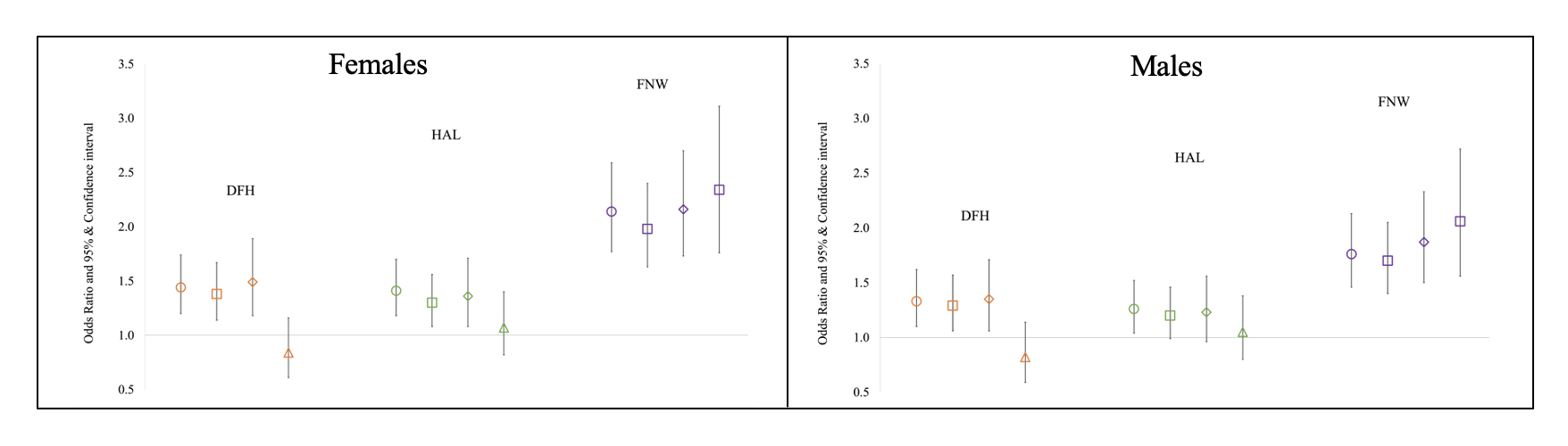


Supplementary Figure 3: Logistic regression results for the associations between geometric parameters – diameter of the femoral head (DFH), hip axis length (HAL) & femoral neck width (FNW) and hospital diagnosed osteoarthritis (HESOA) in sex stratified analyses. Odds ratios with 95% CIs were plotted either side of the points. Circle symbol represents unadjusted analyses (model 1), square indicates adjustment for age and sex (model 2), diamond for age, sex, height and weight (model 3) and triangle for age, sex, height, weight, and remaining geometric parameters (model 4)*.*


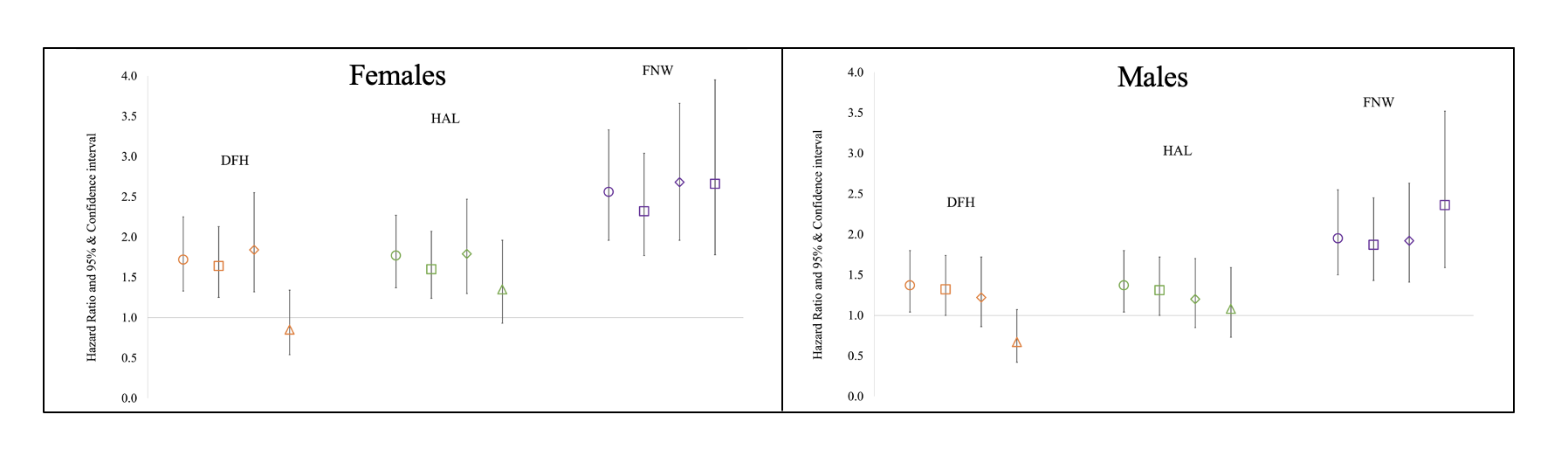


Supplementary Figure 4: Cox regression results for the associations between geometric parameters – diameter of the femoral head (DFH), hip axis length (HAL) & femoral neck width (FNW) and total hip replacement (THR) in sex stratified analyses. Hazard ratios with 95% CIs were plotted either side of the points. Circle symbol represents unadjusted analyses (model 1), square indicates adjustment for age and sex (model 2), diamond for age, sex, height and weight (model 3) and triangle for age, sex, height, weight, and remaining geometric parameters (model 4)*.*

**Supplementary Tables**

Supplementary Table 1: Glossary of terms

| DFH | Diameter of the femoral head |
| --- | --- |
| DXA | Dual-energy x-ray absorptiometry |
| FNW | Femoral neck width |
| HAL | Hip axis length |
| HES | Hospital episode statistics |
| HESOA | Hospital diagnosed osteoarthritis |
| HOA | Hip osteoarthritis |
| HSA | Hip structural analysis |
| OA | Osteoarthritis |
| SSM | Statistical Shape Modelling |
| THR | Total Hip Replacement |

Supplementary Table 2: Comparison between automatically derived GPs and by hip structural analysis.

| Geometric Parameter | **N** | **Hip Structural Analysis** | **Automated Method** | **Correlation R^2^** |
| --- | --- | --- | --- | --- |
|  |  | **Mean [Range]** | **Mean [Range]** |  |
| Femoral Neck Width /Narrowest neck width (NNW) (mm) | 1744 | 35.0 [27.3-49.2] | 31.9 [24.4-42.0] | 0.97 |
| Hip Axis Length (HAL) (mm) | 1689 | 115.1 [91.0-160.9] | 97.0 [77.5-127.3] | 0.93 |

Supplementary Table 3: Logistic regression/ Cox proportional hazard modelling results showing the association between geometric parameters and HOA outcomes in partially adjusted models.

|  | | **Grade ≥ 2 rHOA** | **Grade ≥ 3 rHOA** | **Grade 4 rHOA** | **HESOA** | **THR** |
| --- | --- | --- | --- | --- | --- | --- |
|  |  | **OR [95% CI]** | **OR [95% CI]** | **OR [95% CI]** | **OR [95% CI]** | **HR [95% CI]** |
| Model 1 | FNW | 1.85 [1.78-1.92] | 2.40 [2.22-2.59] | 2.86 [2.43-3.37] | 1.18 [1.09-1.29] | 1.23 [1.09-1.39] |
| Model 2 | FNW | 1.83 [1.73-1.94] | 2.92 [2.62-3.27] | 4.13 [3.31-5.16] | 1.83 [1.60-2.10] | 2.09 [1.73-2.52] |
|  |  |  |  |  |  |  |
| Model 1 | HAL | 1.66 [1.60-1.72] | 1.82 [1.69-1.96] | 1.89 [1.62-2.21] | 1.03 [0.94-1.12] | 1.08 [0.96-1.22] |
| Model 2 | HAL | 1.41 [1.33-1.49] | 1.59 [1.42-1.77] | 1.76 [1.40-2.21] | 1.25 [1.10-1.43] | 1.46 [1.21-1.76] |
|  |  |  |  |  |  |  |
| Model 1 | DFH | 1.58 [1.52-1.64] | 1.77 [1.64-1.90] | 1.90 [1.63-2.22] | 1.04 [0.95-1.13] | 1.06 [0.94-1.20] |
| Model 2 | DFH | 1.27 [1.20-1.34] | 1.51 [1.35-1.69] | 1.82 [1.45-2.30] | 1.33 [1.16-1.53] | 1.47 [1.22-1.78] |

Table shows logistic regression results for the associations between geometric parameters and radiographic hip osteoarthritis (rHOA) ≥ grade 2, ≥ grade 3, grade 4 and hospital diagnosed hip osteoarthritis (HESOA) and cox proportional hazard modelling results between geometric parameters and total hip replacement (THR) in unadjusted and partially adjusted combined sex analyses (models 1 & 2). Model 1 is the unadjusted model and model 2 included the covariates age and sex.

Supplementary Table 4: Logistic regression/ Cox proportional hazard modelling results showing the association between geometric parameters

and HOA outcomes in females.

| **Femoral Neck Width (FNW) (mm)** | **Grade ≥2 rHOA** | **Grade ≥3 rHOA** | **Grade≥ 4 rHOA** | **Hospital diagnosis of HOA** | **THR** |
| --- | --- | --- | --- | --- | --- |
|  | **OR [95% CI]** | **OR [95% CI]** | **OR [95% CI]** | **OR [95% CI]** | **HR [95% CI]** |
| Unadjusted (Model 1) | 2.3 [2.05-2.57] | 4.04 [3.19-5.10] | 6.70 [4.21-10.69] | 2.14 [1.77-2.59] | 2.56 [1.96-3.33] |
| Partially adjusted (Model 2) | 2.20 [1.96-2.47] | 3.73 [2.94-4.73] | 6.20 [3.86-9.98] | 1.98 [1.63-2.40] | 2.32 [1.77-3.04] |
| Partially adjusted (Model 3) | 2.14 [1.87-2.44] | 4.47 [3.39-5.90] | 7.83 [4.48-13.67] | 2.16 [1.73-2.70] | 2.68 [1.96-3.66] |
| Fully adjusted (Model 4) | 2.68 [2.27-3.17] | 6.08 [4.26-8.69] | 8.12 [3.95-16.70] | 2.34 [1.76-3.11] | 2.66 [1.78-3.95] |
| **Hip Axis Length (HAL) (mm)** | | | | | |
| Unadjusted (Model 1) | 1.62 [1.45-1.80] | 2.30 [1.84-2.87] | 3.39 [2.19-5.26] | 1.41 [1.18-1.70] | 1.77 [1.37-2.27] |
| Partially adjusted (Model 2) | 1.54 [1.39-1.72] | 2.10 [1.67-2.63] | 3.08 [1.97-4.80] | 1.30 [1.08-1.56] | 1.60 [1.24-2.07] |
| Partially adjusted (Model 3) | 1.34 [1.17-1.54] | 2.27 [1.71-3.00] | 3.53 [2.03-6.14] | 1.36 [1.08-1.71] | 1.79 [1.30-2.47] |
| Fully adjusted (Model 4) | 1.17 [1.00-1.37] | 1.76 [1.27-2.44] | 2.05 [1.08-3.89] | 1.07 [0.82-1.40] | 1.35 [0.93-1.96] |
| **Diameter of the Femoral Head (DFH) (mm)** | | | | | |
| Unadjusted (Model 1) | 1.53 [1.37-1.71] | 1.93 [1.52-2.44] | 3.26 [2.04-5.21] | 1.44 [1.20-1.74] | 1.72 [1.33-2.25] |
| Partially adjusted (Model 2) | 1.49 [1.33-1.66] | 1.83 [1.44-2.32] | 3.10 [1.93-4.98] | 1.38 [1.14-1.67] | 1.64 [1.25-2.13] |
| Partially adjusted (Model 3) | 1.26 [1.10-1.44] | 1.82 [1.35-2.43] | 3.46 [1.93-6.19] | 1.49 [1.18-1.89] | 1.84 [1.32-2.55] |
| Fully adjusted (Model 4) | 0.62 [0.51-0.75] | 0.42 [0.28-0.64] | 0.62 [0.27-1.42] | 0.84 [0.61-1.16] | 0.85 [0.54-1.34] |

Table shows logistic regression results for the associations between geometric parameters and radiographic hip osteoarthritis (rHOA) ≥ grade 2, ≥ grade 3, grade 4 and hospital diagnosed hip osteoarthritis (HESOA) and cox proportional hazard modelling results between geometric parameters and total hip replacement (THR) in unadjusted, partially and fully adjusted analyses in females (models 1 - 4). Model 1 is the unadjusted model, model 2 included the covariates age and sex, model 3 included model 2 plus height and weight and model 4 is the fully adjusted model including model 3 plus the remaining

geometric parameters.

Supplementary Table 5: Logistic regression/ Cox proportional hazard modelling results showing the association between geometric parameters

and HOA outcomes in males.

| **Femoral Neck Width (FNW) (mm)** | **Grade ≥2 rHOA** | **Grade ≥3 rHOA** | **Grade 4 rHOA** | **Hospital diagnosis of HOA** | **THR** |
| --- | --- | --- | --- | --- | --- |
|  | **OR [95% CI]** | **OR [95% CI]** | **OR [95% CI]** | **OR [95% CI]** | **HR [95% CI]** |
| Unadjusted (Model 1) | 1.73 [1.62-1.85] | 2.74 [2.42-3.1] | 3.76 [2.93-4.83] | 1.76 [1.46-2.13] | 1.95 [1.50-2.55] |
| Partially adjusted (Model 2) | 1.72 [1.61-1.84] | 2.72 [2.40-3.09] | 3.69 [2.87-4.74] | 1.70 [1.40-2.05] | 1.87 [1.43-2.45] |
| Partially adjusted (Model 3) | 1.71 [1.59-1.85] | 3.29 [2.85-3.80] | 4.55 [3.41-6.08] | 1.87 [1.50-2.33] | 1.92 [1.41-2.63] |
| Fully adjusted (Model 4) | 2.29 [2.07-2.53] | 5.00 [4.15-6.04] | 8.61[5.90-12.57] | 2.06 [1.56-2.72] | 2.36 [1.59-3.52] |
| **Hip Axis Length (HAL) (mm)** | | | | | |
| Unadjusted (Model 1) | 1.37 [1.28-1.46] | 1.47 [1.30-1.67] | 1.51 [1.16-1.96] | 1.26 [1.04-1.52] | 1.37 [1.04-1.80] |
| Partially adjusted (Model 2) | 1.36 [1.27-1.45] | 1.45 [1.28-1.65] | 1.46 [1.12-1.90] | 1.20 [0.99-1.46] | 1.31 [1.00-1.72] |
| Partially adjusted (Model 3) | 1.25 [1.15-1.36] | 1.47 [1.25-1.72] | 1.28 [0.91- 1.79] | 1.23 [0.96-1.56] | 1.20 [0.85-1.70] |
| Fully adjusted (Model 4) | 1.27 [1.16-1.40] | 1.27 [1.06-1.52] | 1.08 [0.74-1.57] | 1.05 [0.80-1.38] | 1.08 [0.73-1.59] |
| **Diameter of the Femoral Head (DFH) (mm)** | | | | | |
| Unadjusted (Model 1) | 1.21 [1.13-1.29] | 1.44 [1.27-1.64] | 1.60 [1.23-2.08] | 1.33 [1.10-1.62] | 1.37 [1.04-1.80] |
| Partially adjusted (Model 2) | 1.20 [1.12-1.28] | 1.43 [1.25-1.62] | 1.56 [1.19-2.03] | 1.29 [1.06-1.57] | 1.32 [1.00-1.74] |
| Partially adjusted (Model 3) | 1.03 [0.95-1.12] | 1.41 [1.21-1.65] | 1.41 [1.02-1.96] | 1.35 [1.06-1.71] | 1.22 [0.86-1.72] |
| Fully adjusted (Model 4) | 0.53 [0.47-0.60] | 0.43 [0.34-0.54] | 0.31 [0.19-0.50] | 0.82 [0.59-1.14] | 0.67 [0.42-1.07] |

Table shows logistic regression results for the associations between geometric parameters and radiographic hip osteoarthritis (rHOA) ≥ grade 2, ≥ grade 3, grade 4 and hospital diagnosed hip osteoarthritis (HESOA) and cox proportional hazard modelling results between geometric parameters and total hip replacement (THR) in unadjusted, partially and fully adjusted analyses in males (models 1 - 4). Model 1 is the unadjusted model, model 2 included the covariates age and sex, model 3 included model 2 plus height and weight and model 4 is the fully adjusted model including model 3 plus the remaining geometric parameters.
